# Temporal trends of medical cost and cost-effectiveness in sepsis patients: a Japanese nationwide medical claims database

**DOI:** 10.1186/s40560-022-00624-5

**Published:** 2022-07-14

**Authors:** Takehiko Oami, Taro Imaeda, Taka‑aki Nakada, Toshikazu Abe, Nozomi Takahashi, Yasuo Yamao, Satoshi Nakagawa, Hiroshi Ogura, Nobuaki Shime, Yutaka Umemura, Asako Matsushima, Kiyohide Fushimi

**Affiliations:** 1 Department of Emergency and Critical Care Medicine, Chiba University Graduate School of Medicine, 1-8-1 Inohana, Chuo, Chiba 260-8677 Japan; 2 Health Services Research and Development Center, University of Tsukuba, Tsukuba, Japan; 3 Department of Emergency and Critical Care Medicine, Tsukuba Memorial Hospital, Tsukuba, Japan; 4 Department of Critical Care Medicine, National Center for Child Health and Development, Tokyo, Japan; 5 Department of Traumatology and Acute Critical Medicine, Osaka University Graduate School of Medicine, Osaka, Japan; 6 Department of Emergency and Critical Care Medicine, Graduate School of Biomedical and Health Sciences, Hiroshima University, Hiroshima, Japan; 7 Department of Emergency & Critical Care, Graduate School of Medical Sciences, Nagoya City University, Aichi, Japan; 8 Department of Health Policy and Informatics, Tokyo Medical and Dental University Graduate School of Medical and Dental Sciences, Tokyo, Japan

**Keywords:** Medical cost, Cost-effectiveness, Sepsis, Diagnosis procedure combination, Critical care

## Abstract

**Background:**

Sepsis is the leading cause of death worldwide. Although the mortality of sepsis patients has been decreasing over the past decade, the trend of medical costs and cost-effectiveness for sepsis treatment remains insufficiently determined.

**Methods:**

We conducted a retrospective study using the nationwide medical claims database of sepsis patients in Japan between 2010 and 2017. After selecting sepsis patients with a combined diagnosis of presumed serious infection and organ failure, patients over the age of 20 were included in this study. We investigated the annual trend of medical costs during the study period. The primary outcome was the annual trend of the effective cost per survivor, calculated from the gross medical cost and number of survivors per year. Subsequently, we performed subgroup and multiple regression analyses to evaluate the association between the annual trend and medical costs.

**Results:**

Among 50,490,128 adult patients with claims, a total of 1,276,678 patients with sepsis were selected from the database. Yearly gross medical costs to treat sepsis gradually increased over the decade from $3.04 billion in 2010 to $4.38 billion in 2017, whereas the total medical cost per hospitalization declined (rate = − $1075/year, *p* < 0.0001). While the survival rate of sepsis patients improved during the study period, the effective cost per survivor significantly decreased (rate = − $1806/year [95% CI − $2432 to − $1179], *p* = 0.001). In the subgroup analysis, the trend of decreasing medical cost per hospitalization remained consistent among the subpopulation of age, sex, and site of infection. After adjusting for age, sex (male), number of chronic diseases, site of infection, intensive care unit (ICU) admission, surgery, and length of hospital stay, the admission year was significantly associated with reduced medical costs.

**Conclusions:**

We demonstrated an improvement in annual cost-effectiveness in patients with sepsis between 2010 and 2017. The annual trend of reduced costs was consistent after adjustment with the confounders altering hospital expenses.

**Supplementary Information:**

The online version contains supplementary material available at 10.1186/s40560-022-00624-5.

## Background

Sepsis remains the leading cause of death and a global concern despite the development of medical care [1, 2]. A recent series of demographic studies using international healthcare databases have revealed that sepsis annually affects 48.9 million patients and leads to 11 million deaths, with a global trend of decreasing mortality over the decade [3–6]. To implement effective strategies to accelerate the positive trend of outcomes in sepsis, the accurate estimation of medical costs and resource allocation is urgently needed.

Since the establishment of universal insurance in Japan, an electronic record of the national reimbursement system, called the diagnosis procedure combination (DPC) system, was launched in 2003 to alleviate medical burden and optimize the allocation of medical resources [7, 8]. This system compiles classification files based on the diagnosis and reimbursement for medical costs during hospital stays, which has also been used in numerous epidemiological studies in other fields [9–12]. Using this database, we previously reported an increasing incidence of sepsis and decreased overall mortality from 2010 to 2017 [13], possibly because of the aging society and widespread use of sepsis guidelines.

With the increasing number of sepsis patients, the financial budget for sepsis management has been increasing over the past decade [14]. As further population growth in elderly people is inevitable globally [15], cost-effective strategies for sepsis patients are required to reduce the medical burden. Considering the increasing trend of surviving sepsis, the efficiency of sepsis management is expected to improve despite the growing number of sepsis patients; however, few studies have investigated the annual trend of cost-effectiveness in sepsis. Additionally, the factors increasing the medical budget are yet to be determined. Although severity and comorbidities account for higher medical costs in sepsis patients [16–18], the impact of other variables, such as sex, age, and site of infection on economic outcomes has rarely been clarified. These investigations could provide crucial guidance for determining cost-effective strategies for sepsis management.

Therefore, we hypothesized that cost-effectiveness for sepsis patients has improved over the past decade despite a surge in gross medical costs in Japan. In this study, we investigated the current trend in economic outcomes in sepsis patients using the Japanese nationwide medical claims database from 2010 to 2017.

## Methods

### Study setting and patients

We conducted a retrospective cohort study using the Japanese nationwide medical claims database obtained from the DPC system, which includes diagnoses, interventions, and comorbidities/complications during hospitalization. These classifications are used for medical service reimbursements during acute inpatient care. The DPC data were obtained from 1237 hospitals, which covered 71.5% of acute care facilities in 2017 [8]. All the registered patients were screened for sepsis between 2010 and 2017. Patients over the age of 20 were included in this study without any other exclusion criteria.

This study was approved by the Institutional Review Board of the Chiba University Graduate School of Medicine. The review board waived the need for written informed consent from participants or their guardians.

### Data collection and definition

Using the claims database, the following information was collected: age, sex, length of hospital stay, chronic diseases (malignant tumor, hypertension, diabetes mellitus, heart failure, cerebrovascular disease, ischemic heart disease, chronic respiratory disease, and chronic renal failure), admission to the intensive care unit (ICU), discharge status (home, nursing facility, and inter-hospital transfer), medical cost, site of infection, medical procedures, laboratory tests, and admission diagnosis or complications during hospital stay. Primary diagnosis, comorbidities, and in-hospital complications were recorded as codes based on the International Statistical Classification of Diseases and Related Health Problems 10th revision (ICD-10) (Additional file 1: Table S1). The site of infection was determined according to the following recorded codes: respiratory (mouth, throat, nasal cavity, neck, lung, lower respiratory tract, chest cavity), urogenital (kidney, urinary tract, uterus, genital organs), abdominal (liver, gall bladder, intestine, peritoneal cavity, gastrointestinal system), bone and soft tissue (skin and soft tissue, bone and joint, lymph tissue, breast), meninges/brain/spinal cord, heart, blood, and unknown. Patients with missing data (*n* = 766,395) were excluded from the analysis: only data regarding the site of infection were missing. Multiple codes in the “site of infection” were replaced with the category “Multiple”.

We selected sepsis patients who had presumed serious infection and organ failure during hospital stay [4]. (Additional file 2: Fig. S1). Presumed serious infection was defined as initiation of new antibiotic treatment (intravenous) within ± 2 days followed by blood culture and antibiotic administration for at least consecutive 4 days. Because of unavailability of laboratory data in the database, data regarding organ dysfunction were extracted as follows: use of vasopressors, mechanical ventilation or oxygen supplementation, kidney injury extracted based on diuretics use, diagnostic codes related to kidney dysfunction or renal replacement therapy, liver injury extracted using the codes indicating liver dysfunction, thrombocytopenia, metabolic acidosis.

### Medical cost

The total medical cost per hospitalization includes the fee of drugs, laboratory tests, radiological examinations, and medical procedures during the hospital stay. Although medical fees were mainly reimbursed on a bundled payment basis, we calculated medical costs based on the reference prices in the Japanese fee schedule, as described in a previous report [19]. Since the number of hospitals subject to DPC systems has been increasing over the years, yearly gross medical costs were adjusted by the number of registered patients in the DPC system. Daily medical cost per person was derived by dividing the total medical cost by the length of hospital stay. Furthermore, the value of medical costs was normalized with the consumer price index (CPI) in Japan in 2017 to compare the trend among different years (https://www.e-stat.go.jp/en/dbview?sid=0003427113). The CPI-adjusted costs were calculated using the following formula:

$${\text{CPI}} - {\text{adjusted}}\,{\text{costs}} = {\text{cost}} \times {\text{CPI}}\,{\text{in}}\,2017/{\text{CPI}}\,{\text{in}} \,{\text{the}}\,{\text{admission}}\,{\text{year}}{.}$$

Subsequently, the CPI-adjusted costs were converted into U.S. dollars in accordance with the latest exchange rate between U.S. dollars and Japanese yen as of February 3rd, 2022 (115.25 yen = $1 USD).

### Calculation of cost-effectiveness

As a representative parameter to compare the trend of cost-effectiveness in healthcare settings throughout the study period, we calculated the effective cost per survivor from annual gross medical cost for all patients, including survivors and non-survivors, and number of survivors per year as follows [20, 21]:

$${\text{Effective}}\,{\text{cost}}\,{\text{per}}\,{\text{survivor}} = {\text{annual}}\,{\text{gross}}\,{\text{medical}}\,{\text{cost}}\,{\text{of}}\,{\text{all}}\,{\text{patients}}/{\text{number}}\,{\text{of}}\,{\text{survivors}}\,{\text{per}}\,{\text{year}}{.}$$

### Statistical analysis

The primary outcome was the annual change in the effective cost per survivor in sepsis patients. The significance over different years was analyzed using a linear regression test, on the assumption that the regression plot was followed by linearity. As a sensitivity analysis, we conducted subgroup analyses for cost-effectiveness with regard to age, ICU admission, transfer to other hospitals, site of infection (single or multiple infection), mechanical ventilation, vasopressor therapy, and renal replacement therapy.

The secondary outcomes were the annual trend of medical cost per hospitalization and daily medical cost per person. We then conducted subgroup and multivariable regression analyses to investigate the association between the admission year (independent variable) and medical costs during hospital stay. For the subgroup analysis, sex, age, and site of infection were chosen as the target variables. The age subgroups were divided into adults (20–64 years), early elderly (65–74 years), and late elderly (≥ 75 years), as previously described [13]. Also, the subgroup analysis was conducted based on the site of infection (respiratory, urogenital, abdominal, bone and soft tissue, meninges/brain/spinal cord, heart, blood, multiple, and unknown). To remove baseline imbalances among subgroups, medical costs were adjusted for sex, age, number of chronic diseases, and site of infection using a generalized linear regression model. Using prediction probabilities for target variables, adjusted parameters were expressed with 95% confidence intervals (CI) as representative values in each subgroup. Also, we performed a multiple regression analysis to enhance the robustness of our study. We adjusted medical cost per hospitalization for the following possible confounders: age, sex, site of infection, number of chronic diseases, ICU admission, surgery, and length of hospital stay [16, 22, 23], which were listed on the demographics and clinical characteristics of patients in the cohort. After an evaluation of the association between medical costs and each variable, the variable with a *p* < 0.10 was retained in the regression model. Multicollinearity were measured to check the interaction and confounding among the variables. As a result of regression diagnostics, normal plots of the residuals displayed skewed distribution. Then, we developed a multiple regression analysis using log-transformed costs to better fit the normal distribution. Coefficients calculated from the regression analysis were converted into integer values in the results. Repeated admissions were excluded from mortality analysis.

Data were presented as means (standard deviation), medians (quartiles), or numbers and percentages, as appropriate. A two-tailed *p*-value < 0.05 was considered statistically significant. Data manipulation and statistical analyses were performed using SQL (mariadb v10.4.17), GraphPad Prism 9 (GraphPad Software, San Diego, CA, USA), pandas (v1.0.5), scipy (v1.7.3), numpy (v1.21.4), seaborn (v0.11.2), matplotlib (v3.5.1), and statsmodels (v0.13.2) in Python (v3.9.0).

## Results

### Baseline patient characteristics

Among the 50,490,128 adult patients registered in the DPC system between 2010 and 2017, we included 1,276,678 sepsis patients in this cohort (Additional file 2: Fig. S1). In-hospital mortality was 18.9% during the study period, with a significantly decreasing trend (*p* < 0.0001). The length of hospital stay was decreasing from 34 (17–63) to 26 (14–49) days during the 8 years (rate = − 1.7 days/year, *p* < 0.0001). The median age of this cohort was 77 (67–84). The most frequent site of infection was respiratory (34.6%), followed by multiple (29.2%) and abdominal (13.6%). The average proportion of ICU admission was 15.6%, with the same trend throughout the 8 years (14.8% in 2010 and 14.8% in 2017). The proportion of patients who were transferred to other hospitals was 23.9% during the study period (Additional file 1: Table S2).

### The trend of medical cost

**Fig. 1:**
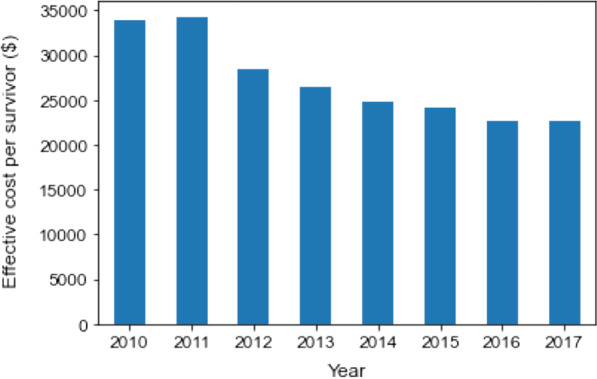
Temporal change in cost-effectiveness in sepsis patients between 2010 and 2017. The bar plot depicts the relationship between the year of hospital admission on the *x*-axis and effective cost per survivor on the *y*-axis. The effective cost per survivor was calculated as follows: the sum of the medical costs of all patients/number of survivors per year. Effective cost per survivor: − $1806/year [95% CI − $2432 to − $1179], *p* = 0.001. The coefficient was calculated using a linear regression analysis

The adjusted yearly gross medical cost gradually increased throughout the study period from $3.04 to $4.38 billion, which was associated with a growing number of sepsis patients (67,318 in 2010 and 233,825 in 2017) (Additional file 3: Fig. S2). By contrast, the average medical cost per hospitalization (rate = − $1075/year, *p* < 0.0001) and daily medical cost per person (rate = − $6.8/year, *p* < 0.0001) declined over the 8 years. Additionally, shorter hospital stay was associated with decreased medical costs (Table 1). As the primary outcome, the effective cost per survivor, indicating the extent of cost-effectiveness, significantly decreased from $33,900 to $22,604 between 2010 and 2017 (rate = − $1806/year [95% CI − $2432 to − $1179], *p* = 0.001) (Fig. 1). As a sensitivity analysis, we conducted subgroup analyses for cost-effectiveness among sepsis patients. The effective cost per survivor in sepsis patients who were transferred to other hospitals was − $1,171/year [95% CI − $2,635 to − $933], *p* = 0.001). Patients with life-sustaining interventions, including mechanical ventilation, vasopressor therapy, and renal replacement therapy, displayed a decreasing trend of the effective cost per survivor during the years. All sensitivity analyses showed similar results to the primary test (Table 2).

**Table 1:** Medical cost and length of hospital stay in sepsis cohort

	Total	Year							
		2010	2011	2012	2013	2014	2015	2016	2017
Number of patients	1,276,678	67,318	100,060	126,414	141,670	181,813	197,388	228,190	233,825
Age, year	77 (67–84)	76 (65–83)	76 (66–83)	75 (65–83)	76 (65–83)	77 (67–84)	77 (67–84)	77 (68–85)	78 (68–85)
Male, *n* (%)	752,275 (58.9)	40,548 (60.2)	60,018 (60.0)	76,476 (60.5)	84,679 (59.8)	106,710 (58.7)	115,588 (58.6)	132,699 (58.2)	135,557 (58.0)
In‐hospital mortality, *n* (%)^a^	216,607 (18.9)	15,620 (24.1)	20,108 (21.7)	24,848 (21.5)	25,405 (19.8)	30,348 (18.6)	31,152 (17.9)	34,801 (17.3)	34,325 (16.9)
Gross medical costs ($)	2.64 × 10^10^	1.73 × 10^9^	2.68 × 10^9^	2.83 × 10^9^	3.01 × 10^9^	3.66 × 10^9^	3.91 × 10^9^	4.28 × 10^9^	4.38 × 10^9^
Adjusted gross medical costs ($)^b^	2.87 × 10^10^	2.36 × 10^9^	3.22 × 10^9^	3.31 × 10^9^	3.55 × 10^9^	3.88 × 10^9^	3.91 × 10^9^	4.02 × 10^9^	4.38 × 10^9^
Total medical cost per hospitalization ($)									
Mean (SD)	20,743 (28,169)	25,742 (26,248)	26,817 (33,192)	22,355 (29,723)	21,237 (27,746)	20,156 (33,135)	19,802 (26,045)	18,738 (25,032)	18,743 (25,442)
Median (IQR)	12,546 (6593–24,597)	17,432 (8795–33,018)	17,033 (8668–33,126)	13,690 (7007–26,699)	12,759 (6629–25,157)	12,092 (6341–23,702)	11,917 (6292–23,219)	11,355 (6144–21,881)	11,410 (6213–21,835)
Daily medical cost per person ($)									
Mean (SD)	510 (410)	568 (332)	574 (619)	493 (395)	495 (364)	501 (486)	505 (388)	499 (349)	504 (354)
Median (IQR)	412 (328–565)	480 (405–617)	485 (412–622)	386 (307–547)	391 (312–550)	396 (319–554)	401 (323–557)	400 (323–550)	404 (326–556)
Length of hospital stay (day)									
Mean (SD)	44.9 (92.9)	50.3 (70.1)	51.6 (106.6)	51.0 (109.8)	47.3 (93.3)	44.2 (86.0)	43.0 (72.1)	41.2 (96.0)	41.1 (99.0)
Median (IQR)	29 (15–54)	34 (17–63)	33 (17–63)	33 (17–61)	30 (16–57)	28 (15–53)	28 (15–52)	26 (15–50)	26 (14–49)

Data are presented as mean (SD) or median (quartile)

*SD* standard deviation, *IQR* interquartile range

^a^After excluding repeat hospitalizations, total number of sepsis patients was 1,143,422

^b^The value was adjusted by the number of registered patients in the DPC system during the year

**Table 2:** Subgroup analyses for cost-effectiveness among sepsis patients

Subgroup	Coefficient ($)	95% CI	*p-*value
Age			
Adults (20–64 years)	− 1635	− 2105 to − 1164	< 0.0001
Early elderly (65–74 years)	− 1892	− 2630 to − 1155	0.001
Late elderly (≥ 75 years)	− 1688	− 2397 to − 980	0.001
ICU			
Yes	− 1784	− 2635 to − 933	0.002
No	− 1799	− 2512 to − 1086	< 0.0001
Transfer to other hospitals			
Yes	− 1171	− 1866 to − 475	0.006
No	− 2001	− 2624 to − 1378	< 0.0001
Site of infection			
Single	− 1628	− 2236 to − 1020	0.001
Multiple	− 2224	− 2866 to − 1582	< 0.0001
Mechanical ventilation			
Yes	− 3182	− 4204 to − 2160	< 0.0001
No	− 1537	− 2206 to − 869	0.001
Vasopressor therapy			
Yes	− 3381	− 4494 to − 2268	< 0.0001
No	− 1690	− 2352 to − 1028	0.001
Renal replacement therapy			
Yes	− 3226	− 4010 to − 2441	< 0.0001
No	− 1661	− 2320 to − 1003	0.001

*CI* confidence interval, *ICU* intensive care unit

### Subgroup analysis

**Fig. 2:**
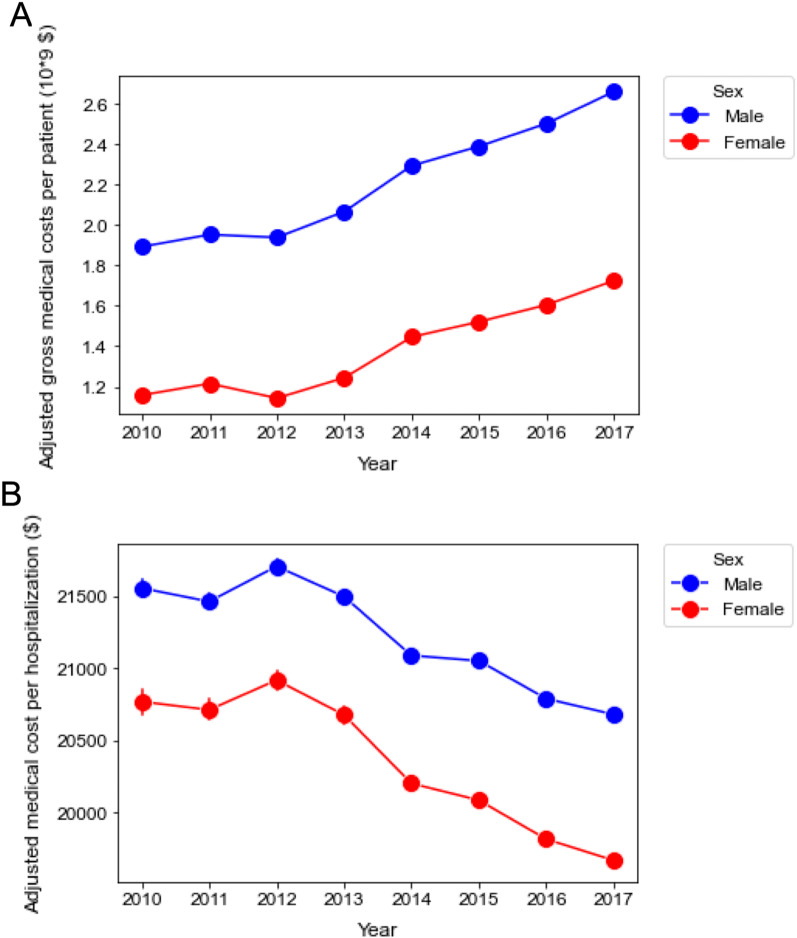
Annual change in medical cost according to sex. **A** Annual changes in adjusted gross medical costs between 2010 and 2017 according to sex. Male: + $115.4 million/year [95% CI $89.5 to $141.0 million], *p* < 0.0001. Female: + $86.2 million/ year [95% CI $58.8 to $114.0 million], *p* < 0.0001. **B** Annual changes in adjusted medical cost per hospitalization between 2010 and 2017 according to sex. Male: − $156/year [95% CI − $163 to − $149], *p* < 0.0001. Female: − $198/year [95% CI − $206 to − $189], *p* < 0.0001. The error bars indicate the 95% confidence interval. The coefficient was calculated using a linear regression analysis

**Fig. 3:**
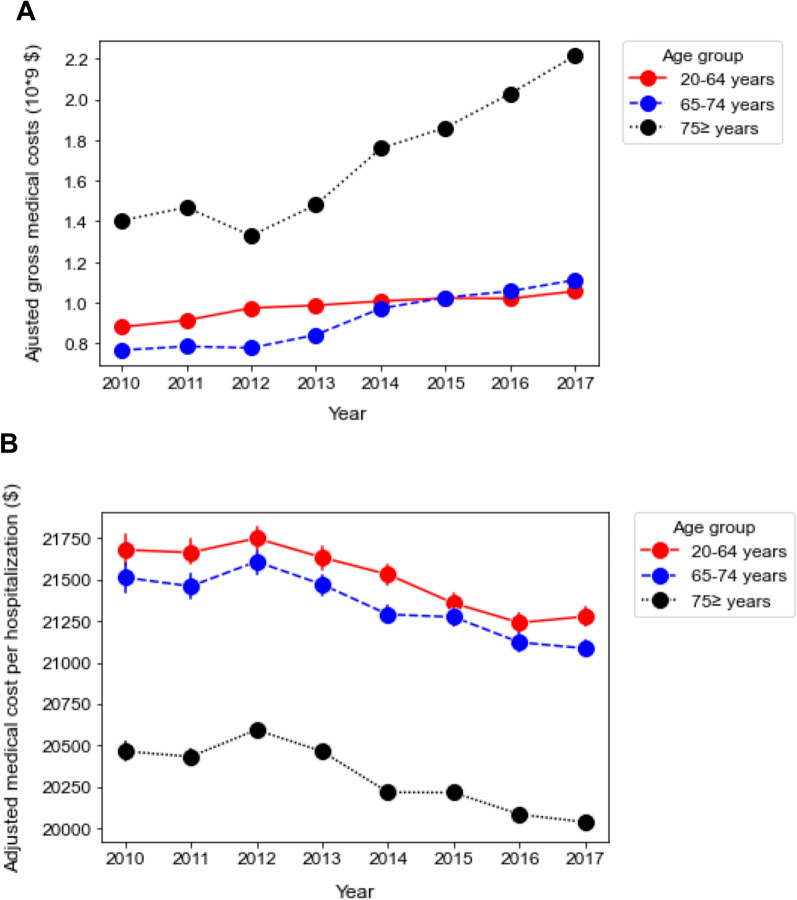
Annual change in medical cost according to age subgroups. **A** Annual changes in adjusted gross medical costs between 2010 and 2017 according to age subgroups. Adults (20 ≤ age ≤ 64): + $23.0 million /year [95% CI − $15.8 to + $30.3 million], *p* < 0.0001. Early elderly (65 ≤ age ≤ 74): + $55.3 million/year [95% CI + $40.9 to + $69.8 million], *p* < 0.0001. Late elderly (75 ≤ age): + $123.2 million/year [95% CI + $76.8 to + $170.0 million], *p* < 0.0001. **B** Annual changes in adjusted medical costs per hospitalization between 2010 and 2017 according to age subgroups. Adults (20 ≤ age ≤ 64): − $81/year [95% CI − $91 to − $70], *p* < 0.0001. Early elderly (65 ≤ age ≤ 74): − $77/year [95% CI − $87 to − $67], *p* < 0.0001. Late elderly (75 ≤ age): − $81/year [95% CI − $86 to − $75], *p* < 0.0001. The error bars indicate the 95% confidence interval. The coefficient was calculated using a linear regression analysis

**Fig. 4:**
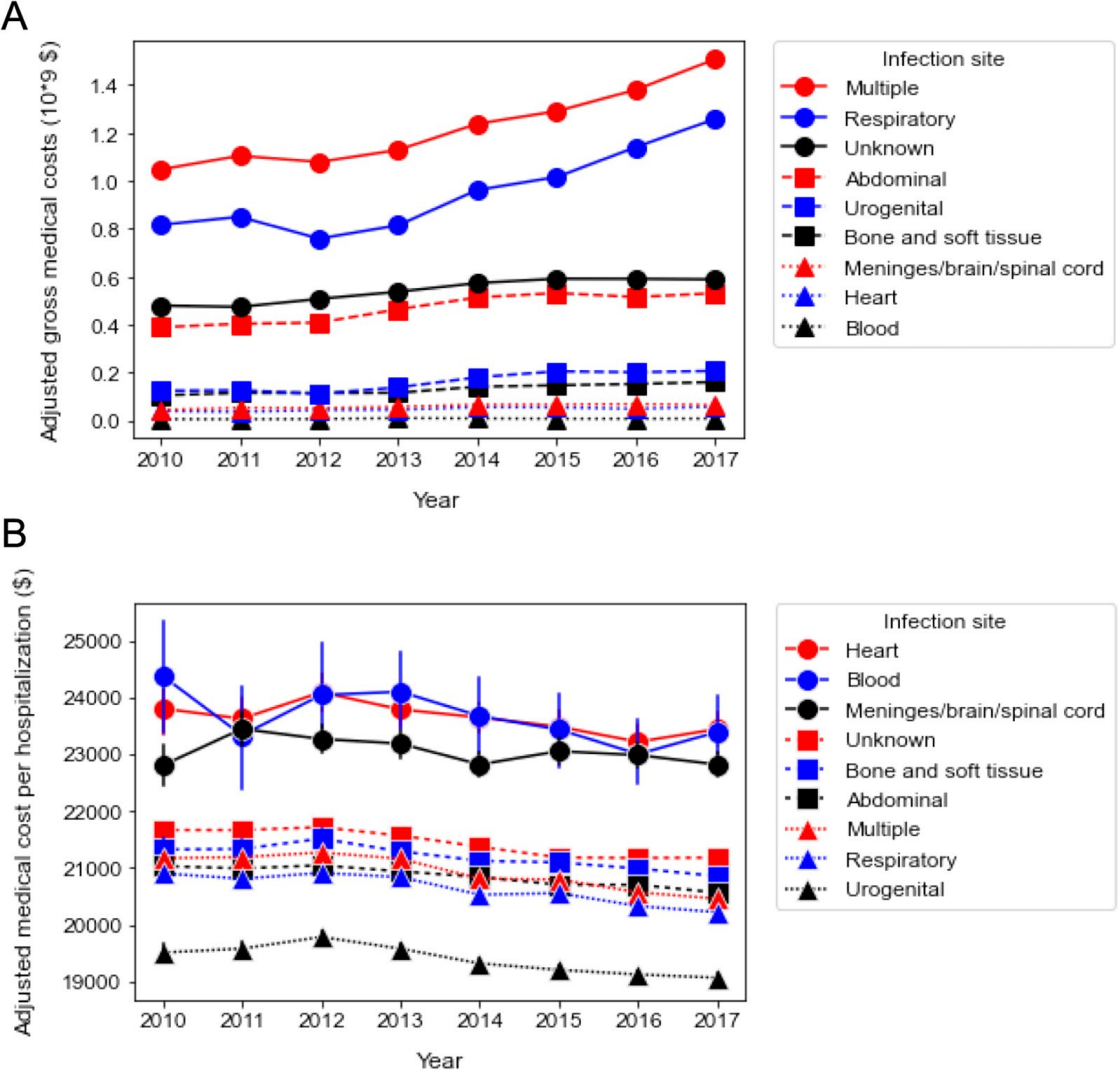
Annual change in medical costs according to site of infection. **A** Annual changes in adjusted gross medical costs between 2010 and 2017 according to site of infection. Multiple: + $63.6 million/year [95% CI + $44.7 to + $82.5 million], *p* < 0.0001. Respiratory: + $65.0 million/year [95% CI + $33.9 to + $96.2 million], *p* = 0.002. Unknown: + $19.5 million/year [95% CI + $13.0 to + $26.1 million], *p* < 0.0001. Abdominal: + $23.4 million/year [95% CI + $14.8 to + $32.1 million], *p* = 0.001. Urogenital: + $15.2 million/year [95% CI + $8.6 to + $21.9 million], *p* = 0.001. Bone and soft tissue: + $8.3 million/year [95% CI + $6.0 to + $10.6], *p* < 0.0001. Meninges/brain/spinal cord: + $3.2 million/year [95% CI + $1.7 to + $4.7 million], *p* = 0.002. Heart: + $2.7 million/year [95% CI + $1.0 to + $4.3 million], *p* = 0.007. Blood: + $0.36 million/year [95% CI − $0.16 to + $0.89 million], *p* = 0.14. **B** Annual changes in adjusted medical costs per hospitalization between 2010 and 2017 according to site of infection. Heart: − $88/year [95% CI − $140 to − $36], *p* = 0.001. Blood: − $135/year [95% CI − $255 to − $15], *p* = 0.027. Meninges/brain/spinal cord: − $52/year [95% CI − $92 to − $12], *p* = 0.010. Unknown: − $96/year [95% CI − $108 to − $83], *p* < 0.0001. Bone and soft tissue: − $87/year [95% CI − $108 to − $65], *p* < 0.0001. Abdominal: − $75/year [95% CI − $85 to − $65], *p* < 0.0001. Multiple: − $130/year [95% CI − $137 to − $122], *p* < 0.0001. Respiratory: − $111/year [95% CI − $117 to − $105], *p* < 0.0001. Urogenital: − $101/year [95% CI − $116 to − $86], *p* < 0.0001. The error bars indicate the 95% confidence interval. The coefficient was calculated using a linear regression analysis

Although the trend of annual gross medical costs and medical costs per hospitalization were comparable between men and women, both parameters were greater in men than in women (Fig. 2, Additional file 4: Fig. S3). In the age subgroups, gross medical cost in the late elderly (≥ 75 years) accounted for a larger proportion (47.3% in 2010 and 50.5% in 2017). The growth rate of the gross medical costs in the later elderly was higher than that in the adults and early elderly over the years (Fig. 3A, Additional file 5: Fig. S4). In contrast to the increasing trend of gross medical costs, the average medical cost per hospitalization gradually decreased with almost the same decrement in the three age subgroups during the study period (Fig. 3, Additional file 5: Fig. S4). In terms of the infection site, multiple infections showed the highest gross medical costs with an increasing trend (rate =  + $63.6 million/year, *p* < 0.0001) followed by respiratory infection (rate = + $65.0 million/year, *p* = 0.002). By contrast, the average medical cost per hospitalization gradually decreased in all infection categories throughout the study period. While heart, blood, and meninge/brain/spinal cord infection presented higher medical costs per person, urogenital infection demonstrated the lowest proportion of medical cost per hospitalization with a shorter hospital stay than the other groups (Fig. 4, Additional file 6: Fig. S5).

### Multiple regression analysis for medical cost

After adjusting for multiple variables, including age, sex (male), site of infection, number of chronic diseases, ICU admission, surgery, and length of hospital stay, the admission year remained a significant association to reduce medical costs (Table 3).

**Table 3:** Multivariable regression analysis of medical cost

Variable	Coefficient^a^	95% CI	*p-*value
Age -per year	0.998	0.997–0.999	< 0.0001
Sex (male)	1.047	1.044–1.049	< 0.0001
Number of chronic diseases	1.078	1.075–1.081	< 0.0001
ICU admission	1.740	1.733–1.745	0.0001
Surgery	2.138	2.133–2.142	
Length of hospital stay -per day	1.003	1.002–1.004	< 0.0001
Site of infection			
Abdominal	Reference		< 0.0001
Blood	2.028	1.954–2.103	< 0.0001
Bone and soft tissue	1.186	1.177–1.193	< 0.0001
Heart	1.620	1.595–1.644	< 0.0001
Meninges/brain/spinal cord	1.482	1.465–1.499	< 0.0001
Respiratory	1.096	1.091–1.101	< 0.0001
Urogenital	1.049	1.044–1.056	< 0.0001
Multiple	1.324	1.321–1.330	< 0.0001
Unknown	1.419	1.412–1.425	< 0.0001
Admission year	0.966	0.966–0.968	< 0.0001

*CI* confidence interval, *ICU* intensive care unit

^a^100 × (coefficient − 1) shows percent change in medical costs

## Discussion

In this study, we demonstrated an improvement in annual cost-effectiveness in sepsis patients using the Japanese nationwide medical claims database. In addition, the annual trend of reduced costs was consistent after confounding adjustment with age, sex (male), infection sites, number of chronic diseases, ICU admission, surgery, and length of hospital stay.

The gross medical cost of sepsis treatment has surged with an increasing number of patients over the past decade [14]. An analysis of paid Medicare claims in the U.S. showed that 20 billion dollars were spent annually to treat one million sepsis patients, with an increasing trend from 2012 to 2018. The increasing trend in medical expenses is presumably due to population growth, especially among the older generation. In terms of medical cost per hospitalization, a French study reported a decreasing trend in the median hospitalization expenses for septic shock from €17,261 in 2010 to €16,365 in 2017 [24]. The budget reduction might be attributed to the shortened length of stay and the high proportion of elderly people who are likely to be placed in limited care or transferred to rehabilitation. By contrast, another study conducted in Brazil reported a gradual increase in the mean medical cost per case from $512.6 in 2006 to $619.2 in 2014, with an increase in the number of sepsis patients [25]. These data implicate a wide variability of economic outcomes in sepsis, depending on the reimbursement system and economic structure [26]. Among Asian countries without sufficient reports about the medical costs of sepsis treatment, our study provides meaningful insight. The present study using the Japanese database demonstrated decreasing medical cost per hospitalization with shortened length of stay, suggesting that an improvement in survival rate potentially contributed to alleviating the economic burden.

Although the temporal trend of cost-effectiveness has rarely been investigated in sepsis patients, we demonstrated a significant decrease in the effective cost per survivor through the study period. To alleviate the distribution of medical resources, a precise estimation of the efficiency of the healthcare system is required. As an optimal way to assess the medical burden, cost-effectiveness is a reliable parameter [27]. In a previous study investigating cost-effectiveness using a cohort of critically ill patients, the parameter clearly illustrated an improvement in the efficiency of medical allocation with an increasing survival rate during ICU admission [21, 28, 29]. In another study, cost-effectiveness with total medical cost and quality-adjusted life years was used to estimate the relationship between medical cost and resource use [30, 31]. In our study, we demonstrated an improvement in cost-effectiveness as well as a decrease in medical costs per hospitalization and a shortened duration of hospital stay. A plausible reason to support this trend is the widespread use of guidelines and the improvement in the quality of acute care [32, 33]. Also, the DPC system might have contributed to decreasing duration of hospital stay, leading to cost-effective medical care [34, 35]. Further clarification is warranted to examine the elements responsible for explaining this trend. As the number of elderly people is expected to increase exponentially in Japan, efficient strategies to improve cost-effectiveness are required to reduce the medical burden in the future [36].

Several variables have been reportedly related to a surge in the medical costs of patients with sepsis [22]. A recent retrospective observational study reported a proportional increase in medical costs according to the severity of sepsis and a higher economic burden in sepsis cases that were not diagnosed at admission [16]. Another study clarified that comorbidities and acuity of illness had a positive impact on increasing medical expenses for sepsis patients [17]. In addition, cancer patients who were diagnosed with sepsis incurred an additional cost of $29,081, which is twice the burden of cancer care costs [18]. In our study, not only ICU admission and chronic diseases but also sex, age, and site of infection contributed to increasing medical costs.

In general, female patients have greater healthcare expenditures, possibly due to a higher number of chronic diseases compared with male patients [37–39]. By contrast, our study demonstrated that male sepsis patients had higher medical costs than female patients. Gender differences in sepsis might contribute to higher mortality and a longer duration of hospital stay in male patients [40]. It is plausible that older patients need more medical resources than younger patients [41], but the late elderly showed the least medical cost despite having the highest gross medical cost among the three age subgroups. This discrepancy might be explained by the fact that the intensity of treatment, such as ICU admission or artificial organ support, is likely lower in elderly patients than in adult patients [42, 43]. While medical staffs are unlikely to select more life-sustaining treatment on older patients, a widespread advanced care planning has potentially contributed to promoting the decision-making [44, 45]. In addition, the increasing population of 80- and 90-year-olds, which characterizes the first super-aging society, is potentially attributed to the surge in medical costs [36].

Although the economic burden of healthcare-associated infections has been mentioned before [46], few studies have investigated the impact of infection sites on medical costs in sepsis patients. While respiratory and urogenital infections were associated with lower medical costs, other categories of infection, such as blood, heart, brain, and spinal were positively associated with medical expenses. These types of infections prolong the duration of treatment during hospitalization and deteriorate clinical consequences [47]; therefore, the duration of hospital stay presumably affects the total medical cost in sepsis patients. Further investigations are needed to validate the significant contribution of these variables to changes in medical costs.

This study had several limitations. First, it was a retrospective study; however, the number of enrolled patients was substantial. Second, the latest definition of sepsis, life-threatening organ dysfunction caused by a dysregulated host response to infection, was not applied in this cohort because of the lack of laboratory results. The validity of our method is supported by a previous report that medical claims data could be used for analysis, similar to conventionally collected data [4]. Third, the value of the medical cost potentially includes unrelated payments other than sepsis treatment, since some sepsis patients were primarily hospitalized for other diagnostic diseases. Fourth, we replaced multiple codes regarding the site of infection using the “Multiple” category (29.2%), which might have blurred crucial features regarding organ-specific infection. Fifth, the length of hospital stay in Japan is longer than in other countries [13], which could decrease the generalizability of the present results. Sixth, long-term mortality or quality-adjusted life years were not investigated because of the lack of available connections between our cohort and other healthcare databases. In this regard, a record linking different databases would provide us with more comprehensive and elaborate information on sepsis patients.

## Conclusions

In this study, using the Japanese nationwide medical claims database, we demonstrated an improvement in cost-effectiveness in sepsis patients over the decade. The annual trend of reduced hospital expenses remained consistent after confounding adjustment.

## Supplementary Information

**Additional file 1: Table S1.** Diagnostic categories with corresponding ICD-10. **Table S2.** Demographics and clinical characteristics of patients with sepsis in the cohort

**Additional file 2: Figure S1.** Flowchart of study population

**Additional file 3: Figure S2.** Temporal changes in gross medical costs and number of sepsis patients between 2010 and 2017

**Additional file 4: Figure S3.** Annual changes in medical costs and length of hospital stay according to sex

**Additional file 5: Figure S4.** Annual changes in medical costs and length of hospital stay according to age subgroups

**Additional file 6: Figure S5.** Annual changes in medical costs and length of hospital stay according to the site of infection.

## Data Availability

The datasets used and analyzed in our study are available from the corresponding author upon reasonable request.

## Abbreviations

DPC: Diagnosis procedure combination
ICU: Intensive care unit
OR: Odds ratio
CI: Confidence interval

## References

[1] Singer M, Deutschman CS, Seymour CW, Shankar-Hari M, Annane D, Bauer M (2016). The Third International Consensus Definitions for Sepsis and Septic Shock (Sepsis-3). JAMA.

[2] Rudd KE, Johnson SC, Agesa KM, Shackelford KA, Tsoi D, Kievlan DR (2020). Global, regional, and national sepsis incidence and mortality, 1990–2017: analysis for the Global Burden of Disease Study. Lancet.

[3] Fleischmann C, Scherag A, Adhikari NK, Hartog CS, Tsaganos T, Schlattmann P (2016). Assessment of global incidence and mortality of hospital-treated sepsis. Current estimates and limitations. Am J Respir Crit Care Med.

[4] Rhee C, Dantes R, Epstein L, Murphy DJ, Seymour CW, Iwashyna TJ (2017). Incidence and Trends of Sepsis in US Hospitals Using Clinical vs Claims Data, 2009–2014. JAMA.

[5] Fleischmann-Struzek C, Mellhammar L, Rose N, Cassini A, Rudd KE, Schlattmann P (2020). Incidence and mortality of hospital- and ICU-treated sepsis: results from an updated and expanded systematic review and meta-analysis. Intensive Care Med.

[6] Bauer M, Gerlach H, Vogelmann T, Preissing F, Stiefel J, Adam D (2020). Mortality in sepsis and septic shock in Europe, North America and Australia between 2009 and 2019- results from a systematic review and meta-analysis. Crit Care.

[7] Yamana H, Moriwaki M, Horiguchi H, Kodan M, Fushimi K, Yasunaga H (2017). Validity of diagnoses, procedures, and laboratory data in Japanese administrative data. J Epidemiol.

[8] Hayashida K, Murakami G, Matsuda S, Fushimi K (2021). History and Profile of Diagnosis Procedure Combination (DPC): development of a real data collection system for acute inpatient care in Japan. J Epidemiol.

[9] Moroi R, Tarasawa K, Shiga H, Yano K, Shimoyama Y, Kuroha M (2021). Efficacy of urgent colonoscopy for colonic diverticular bleeding: a propensity score-matched analysis using a nationwide database in Japan. J Gastroenterol Hepatol.

[10] Mine Y, Fujino Y, Sabanai K, Muramatsu K, Otani M, Kubo T (2020). Effectiveness of regional clinical pathways on postoperative length of stay for hip fracture patients: a retrospective observational study using the Japanese Diagnosis Procedure Combination database. J Orthop Sci.

[11] Kobori S, Kubo T, Otani M, Muramatsu K, Fujino Y, Adachi H (2017). Coexisting infectious diseases on admission as a risk factor for mechanical ventilation in patients with Guillain-Barre syndrome. J Epidemiol.

[12] Shinjo D, Matsumoto K, Terashima K, Takimoto T, Ohnuma T, Noguchi T (2019). Volume effect in paediatric brain tumour resection surgery: analysis of data from the Japanese national inpatient database. Eur J Cancer.

[13] Imaeda T, Nakada TA, Takahashi N, Yamao Y, Nakagawa S, Ogura H (2021). Trends in the incidence and outcome of sepsis using data from a Japanese nationwide medical claims database-the Japan Sepsis Alliance (JaSA) study group. Crit Care.

[14] Buchman TG, Simpson SQ, Sciarretta KL, Finne KP, Sowers N, Collier M (2020). Sepsis Among Medicare Beneficiaries: 1. The Burdens of Sepsis, 2012–2018. Crit Care Med.

[15] The Lancet Healthy L (2021). Care for ageing populations globally. Lancet Healthy Longev..

[16] Paoli CJ, Reynolds MA, Sinha M, Gitlin M, Crouser E (2018). Epidemiology and costs of sepsis in the united states-an analysis based on timing of diagnosis and severity level. Crit Care Med.

[17] Lee H, Doig CJ, Ghali WA, Donaldson C, Johnson D, Manns B (2004). Detailed cost analysis of care for survivors of severe sepsis. Crit Care Med.

[18] Tew M, Dalziel K, Thursky K, Krahn M, Abrahamyan L, Morris AM (2021). Excess cost of care associated with sepsis in cancer patients: results from a population-based case-control matched cohort. PLoS ONE.

[19] Endo A, Shiraishi A, Otomo Y, Fushimi K, Murata K (2019). Volume-outcome relationship on survival and cost benefits in severe burn injury: a retrospective analysis of a Japanese nationwide administrative database. J Intensive Care.

[20] Reynolds HN, Haupt MT, Thill-Baharozian MC, Carlson RW (1988). Impact of critical care physician staffing on patients with septic shock in a university hospital medical intensive care unit. JAMA.

[21] Raj R, Bendel S, Reinikainen M, Hoppu S, Luoto T, Ala-Kokko T (2018). Temporal trends in healthcare costs and outcome following ICU admission after traumatic brain injury. Crit Care Med.

[22] Hajj J, Blaine N, Salavaci J, Jacoby D (2018). The, “Centrality of Sepsis”: a review on incidence, mortality, and cost of care. Healthcare (Basel)..

[23] Yasui H, Michihata N, Matsui H, Fushimi K, Iwase S, Yoshiuchi K (2021). Association between ambulance use and hospitalization costs among heart failure patients. Heart Vessels.

[24] Dupuis C, Bouadma L, Ruckly S, Perozziello A, Van-Gysel D, Mageau A (2020). Sepsis and septic shock in France: incidences, outcomes and costs of care. Ann Intensive Care.

[25] Quintano Neira RA, Hamacher S, Japiassu AM (2018). Epidemiology of sepsis in Brazil: incidence, lethality, costs, and other indicators for Brazilian Unified Health System hospitalizations from 2006 to 2015. PLoS ONE.

[26] Chalupka AN, Talmor D (2012). The economics of sepsis. Crit Care Clin.

[27] Wilcox ME, Vaughan K, Chong C, Neumann PJ, Bell CM (2019). Cost-effectiveness studies in the ICU: a systematic review. Crit Care Med.

[28] Edbrooke DL, Minelli C, Mills GH, Iapichino G, Pezzi A, Corbella D (2011). Implications of ICU triage decisions on patient mortality: a cost-effectiveness analysis. Crit Care.

[29] Efendijev I, Folger D, Raj R, Reinikainen M, Pekkarinen PT, Litonius E (2018). Outcomes and healthcare-associated costs one year after intensive care-treated cardiac arrest. Resuscitation.

[30] Jukarainen S, Mildh H, Pettila V, Hakkinen U, Peltola M, Ala-Kokko T (2020). Costs and cost-utility of critical care and subsequent health care: a multicenter prospective study. Crit Care Med.

[31] Lindemark F, Haaland OA, Kvale R, Flaatten H, Norheim OF, Johansson KA (2017). Costs and expected gain in lifetime health from intensive care versus general ward care of 30,712 individual patients: a distribution-weighted cost-effectiveness analysis. Crit Care.

[32] Nguyen HB, Corbett SW, Steele R, Banta J, Clark RT, Hayes SR (2007). Implementation of a bundle of quality indicators for the early management of severe sepsis and septic shock is associated with decreased mortality. Crit Care Med.

[33] Egi M, Ogura H, Yatabe T, Atagi K, Inoue S, Iba T (2021). The Japanese Clinical Practice Guidelines for Management of Sepsis and Septic Shock 2020 (J-SSCG 2020). Acute Med Surg.

[34] Hamada H, Sekimoto M, Imanaka Y (2012). Effects of the per diem prospective payment system with DRG-like grouping system (DPC/PDPS) on resource usage and healthcare quality in Japan. Health Policy.

[35] Kondo A, Kawabuchi K (2012). Evaluation of the introduction of a diagnosis procedure combination system for patient outcome and hospitalisation charges for patients with hip fracture or lung cancer in Japan. Health Policy.

[36] McCurry J (2015). Japan will be model for future super-ageing societies. Lancet.

[37] Owens GM (2008). Gender differences in health care expenditures, resource utilization, and quality of care. J Manag Care Pharm.

[38] Bertakis KD, Azari R (2010). Patient gender differences in the prediction of medical expenditures. J Womens Health (Larchmt).

[39] Cohen SB, Ezzati-Rice T, Yu W (2006). The utility of extended longitudinal profiles in predicting future health care expenditures. Med Care.

[40] Xu J, Tong L, Yao J, Guo Z, Lui KY, Hu X (2019). Association of sex with clinical outcome in critically Ill sepsis patients: a retrospective analysis of the large clinical database MIMIC-III. Shock.

[41] McCabe JJ, Cournane S, Byrne D, Conway R, O'Riordan D, Silke B (2017). Age and the economics of an emergency medical admission-what factors determine costs?. QJM.

[42] Peigne V, Somme D, Guerot E, Lenain E, Chatellier G, Fagon JY (2016). Treatment intensity, age and outcome in medical ICU patients: results of a French administrative database. Ann Intensive Care.

[43] Boumendil A, Aegerter P, Guidet B, Network CU-R (2005). Treatment intensity and outcome of patients aged 80 and older in intensive care units: a multicenter matched-cohort study. J Am Geriatr Soc.

[44] Miyashita J, Kohno A, Shimizu S, Kashiwazaki M, Kamihiro N, Okawa K (2021). Healthcare providers’ perceptions on the timing of initial advance care planning discussions in Japan: a mixed-methods study. J Gen Intern Med.

[45] Yamamoto K, Yonekura Y, Nakayama K (2022). Healthcare providers’ perception of advance care planning for patients with critical illnesses in acute-care hospitals: a cross-sectional study. BMC Palliat Care.

[46] Stone PW (2009). Economic burden of healthcare-associated infections: an American perspective. Expert Rev Pharmacoecon Outcomes Res.

[47] Abe T, Ogura H, Kushimoto S, Shiraishi A, Sugiyama T, Deshpande GA (2019). Variations in infection sites and mortality rates among patients in intensive care units with severe sepsis and septic shock in Japan. J Intensive Care.

