# Supplementary material for: Temporal trends of medical cost and cost-effectiveness in sepsis patients: a Japanese nationwide medical claims database
Source: J Intensive Care. 2022 Jul 14;10:33. doi: 10.1186/s40560-022-00624-5 (PMC9281011; doi:10.1186/s40560-022-00624-5)
Supplement: Supplementary file 1 — Additional file 1: Table S1. Diagnostic categories with corresponding ICD-10. Table S2. Demographics and clinical characteristics of patients with sepsis in the cohort [file 40560_2022_624_MOESM1_ESM.pdf]

Temporal trends of medical cost and cost effectiveness in sepsis patients - a Japanese nationwide medical claims database

Authors: Takehiko Oami[1], Taro Imaeda[1], Taka-aki Nakada[1], Toshikazu Abe[2,3], Nozomi Takahashi[1], Yasuo Yamao[1], Satoshi Nakagawa[4], Hiroshi Ogura[5], Nobuaki Shime[6], Yutaka Umemura[5], Asako Matsushima[7] and Kiyohide Fushimi[8]

Affiliations:

1. Department of Emergency and Critical Care Medicine, Chiba University Graduate School of Medicine, Chiba, Japan
2. Health Services Research and Development Center, University of Tsukuba, Tsukuba, Japan.
3. Department of Emergency and Critical Care Medicine, Tsukuba Memorial Hospital, Tsukuba, Japan.
4. Department of Critical Care Medicine, National Center for Child Health and Development, Tokyo, Japan
5. Department of Traumatology and Acute Critical Medicine, Osaka University Graduate School of Medicine, Osaka, Japan
6. Department of Emergency and Critical Care Medicine, Graduate School of Biomedical and Health Sciences, Hiroshima University, Hiroshima, Japan
7. Department of Advancing Acute Medicine, Graduate School of Medical Sciences, Nagoya City University, Aichi, Japan
8. Department of Health Policy and Informatics, Tokyo Medical and Dental University Graduate School of Medical and Dental Sciences, Tokyo, Japan.

Correspondence:

Taka-aki Nakada

Chiba University Graduate School of Medicine, Department of Emergency and Critical Care Medicine, 1-8-1 Inohana, Chuo, Chiba 260-8677, Japan

Phone: +81-43-226-2372, Fax: +81-43-226-2371,

Email: taka.nakada@nifty.com

Additional files

Additional File 1:
Table S1. Diagnostic categories with corresponding ICD-10

Table S2. Demographics and clinical characteristics of patients with sepsis in the cohort

Additional File 2: Figure S1. Flowchart of study population

Additional File 3: Figure S2. Temporal changes in gross medical costs and number of sepsis patients between 2010 and 2017

Additional File 4: Figure S3. Annual changes in medical costs and length of hospital stay according to sex

Additional File 5: Figure S4. Annual changes in medical costs and length of hospital stay according to age subgroups

Additional File 6: Figure S5. Annual changes in medical costs and length of hospital stay according to the site of infection

List of abbreviations

DPC, diagnosis procedure combination; ICU, intensive care unit; OR, odds ratio; CI, confidence interval.

Table S1 Diagnostic categories with corresponding ICD-10

Diagnosis	ICD-10 codes
Comorbidity	
Malignant tumor	C00-C97, D00-D09
Hypertension	I10-I15
Diabetes mellitus	E10-E14
Heart failure	I50
Ischemic heart disease	I60-I69
Cerebrovascular disease	I20-I25
Chronic respiratory disease	J40-J47
Chronic renal failure	N18
Focus of infection	
Respiratory	A15-A16, J00-J06, J09-J18, J20-J22, J31-J32, J35-J37, J39.0, J39.1, J85-J86
Urogenital	A18.1, A51.0, A54.0-A54.2, A56.0-A56.2, A59.0, A60.0, N30.0, N30.8, N39.0, N41.0-N41.3, N45, N49.0-N49.2, N70-N77, O23
Abdominal	A00-A09, A18.3, A42.1, A74.8, K35-K38, K57.0, K57.2, K57.4, K57.8, K61, K63.0, K63.1, K65, K67, K75.0, K80.0, K80.1, K80.3, K80.4, K81, K83.0
Bone and soft tissue	A18.0, A18.4, A26.0, A28.1, A31.1, A31.8, A32.0, A36.3, A42.2, A43.1, A46, A48.0, L00-L08, M00, M01.0, M46.3, M46.5, M49.1-M49.3, M60.0, M86.0, M86.1, M86.65, M86.66, M86.69, M86.99
Blood	A19, A40.0, A49.0, A49.1, A49.9
Organ dysfunction	
Renal	N00.9, N10, N17.0, N17.1, N17.8, N17.9
Hepatic	K72.0, K72.9, K76.8
Thrombocytopenia	D69.5, D69.6
Coagulopathy	D65, D68.9
Acidosis	E87.2

Table S2 Demographics and clinical characteristics of patients with sepsis in the cohort

	Total	Year							
		2010	2011	2012	2013	2014	2015	2016	2017
Number of patients	1,276,678	67,318	100,060	126,414	141,670	181,813	197,388	228,190	233,825
Community-onset sepsis, n (%)	739,877 (58.0)	34,346 (51.0)	51,693 (51.6)	65,261 (51.6)	78,923 (55.7)	107,781 (59.2)	119,387 (60.4)	139,354 (61.0)	143,132 (61.2)
Comorbidity									
Malignant tumor, n (%)	417,735 (32.7)	22,989 (34.1)	33,734 (33.7)	44,188 (35.0)	48,715 (34.4)	59,489 (32.7)	63,630 (32.2)	71,333 (31.3)	73,657 (31.5)
Hypertension, n (%)	323,936 (25.4)	15,460 (23.0)	23,929 (23.9)	29,979 (23.7)	34,630 (24.4)	46,254 (25.4)	49,522 (25.1)	60,911 (26.7)	63,251 (27.1)
Diabetes mellitus, n (%)	277,203 (21.7)	14,006 (20.8)	21,642 (21.6)	26,714 (21.1)	30,360 (21.4)	39,190 (21.6)	43,321 (21.9)	49,974 (21.9)	51,996 (22.2)
Heart failure, n (%)	235,929 (18.5)	12,269 (18.2)	19,046 (19.0)	22,658 (17.9)	25,149 (17.8)	33,052 (18.2)	36,738 (18.6)	42,511 (18.6)	44,506 (19.0)
Cerebrovascular disease, n (%)	175,756 (13.8)	9,302 (13.8)	14,058 (14.0)	17,128 (13.5)	19,066 (13.5)	25,423 (14.0)	27,286 (13.8)	31,436 (13.8)	32,057 (13.7)
Ischemic heart disease, n (%)	120,451 (9.4)	6,362 (9.5)	9,540 (9.5)	12,031 (9.5)	13,377 (9.4)	17,120 (9.4)	18,358 (9.3)	21,604 (9.5)	22,059 (9.4)
Chronic respiratory disease, n (%)	130,052 (10.2)	6,710 (10.0)	10,172 (10.2)	12,257 (9.7)	14,234 (10.0)	19,236 (10.6)	20,922 (10.6)	23,312 (10.2)	23,209 (9.9)
Chronic renal failure, n (%)	47,991 (3.8)	2,874 (4.3)	4,140 (4.1)	5,190 (4.1)	5,491 (3.9)	7,300 (4.0)	7,444 (3.8)	7,863 (3.4)	7,689 (3.3)
Site of infection									
Respiratory, n (%)	442,243 (34.6)	22,636 (33.6)	34,196 (34.1)	40,848 (32.3)	46,054 (32.5)	61,766 (33.9)	66,844 (33.8)	83,071 (36.4)	86,828 (37.1)
Urogenital, n (%)	82,849 (6.4)	3,724 (5.5)	5,549 (5.5)	6,970 (5.5)	8,898 (6.2)	13,044 (7.1)	14,863 (7.5)	15,216 (6.6)	14,585 (6.2)
Abdominal, n (%)	174,340 (13.6)	9,479 (14.0)	13,737 (13.7)	18,593 (14.7)	21,424 (15.1)	25,868 (14.2)	28,096 (14.2)	28,525 (12.5)	28,618 (12.2)

Bone and soft tissue, n (%)	45,778 (3.5)	2,309 (3.4)	3,545 (3.5)	4,538 (3.5)	4,932 (3.4)	6,882 (3.7)	7,522 (3.8)	8,117 (3.5)	7,933 (3.3)
Meninges/brain/ spinal cord, n (%)	14,658 (1.1)	819 (1.2)	1,200 (1.1)	1,595 (1.2)	1,845 (1.3)	2,275 (1.2)	2,384 (1.2)	2,387 (1.0)	2,153 (0.9)
Heart, n (%)	8,538 (0.6)	526 (0.7)	728 (0.7)	972 (0.7)	1,019 (0.7)	1,330 (0.7)	1,330 (0.6)	1,299 (0.5)	1,334 (0.5)
Blood, n (%)	1,281 (0.1)	59 (0.08)	95 (0.09)	129 (0.1)	181 (0.1)	191 (0.1)	187 (0.09)	221 (0.09)	218 (0.09)
Multiple, n (%)	373,829 (29.2)	19,972 (29.6)	29,954 (29.9)	37,974 (30.0)	41,394 (29.2)	51,235 (28.1)	54,994 (27.8)	67,328 (29.5)	70,978 (30.3)
Unknown, n (%)	133,162 (10.4)	7,794 (11.5)	11,056 (11.0)	14,795 (11.7)	15,923 (11.2)	19,222 (10.5)	21,168 (10.7)	22,026 (9.6)	21,178 (9.0)
Length of antibiotic treatment, days									
Mean (SD)	20.2 (22.9)	19.6 (19.8)	19.8 (21.3)	20.8 (23.9)	20.5 (24.0)	19.9 (22.1)	20.0 (22.6)	20.3 (23.0)	20.5 (24.0)
Median (IQR)	13 (8-23)	13 (8-23)	13 (8-23)	14 (8-24)	13 (8-24)	13 (8-23)	13 (8-23)	13 (8-23)	13 (8-23)
Length of hospital stay, days									
Mean (SD)	44.9 (92.9)	50.4 (70.2)	51.7 (106.7)	51.1 (109.9)	47.3 (93.3)	44.3 (86.1)	43.0 (72.1)	41.2 (96.1)	41.2 (99.0)
Median (IQR)	29 (15-54)	34 (17-63)	33 (17-63)	33 (17-61)	30 (16-57)	28 (15-53)	28 (15-52)	26 (15-50)	26 (14-49)
ICU admission, n (%)	199,766 (15.6)	9,941 (14.8)	14,968 (15.0)	21,798 (17.2)	24,111 (17.0)	29,576 (16.3)	30,858 (15.6)	34,002 (14.9)	34,512 (14.8)
Surgery, n (%)	570,299 (44.7)	32,346 (48.0)	47,191 (47.2)	62,003 (49.0)	66,991 (47.3)	80,882 (44.5)	85,446 (43.3)	95,988 (42.1)	99,452 (42.5)
Discharge status [a]									
Home	698,214 (67.9)	36,604 (72.4)	55,343 (71.8)	68,852 (70.5)	78,325 (70.2)	100,183 (67.9)	108,734 (67.3)	123,722 (65.8)	126,451 (65.3)
Nursing facility	77,225 (7.5)	2,051 (4.1)	3478 (4.5)	4,298 (4.4)	5,284 (4.7)	12,368 (8.4)	13,825 (8.6)	17,606 (9.4)	18,315 (9.5)

Inter-hospital transfer	245,635 (23.9)	11,086 (21.9)	16,819 (21.8)	22,897 (23.4)	26,260 (23.5)	34,654 (23.5)	38,704 (24.0)	46,511 (24.7)	48,704 (25.1)
Others	6,765 (0.7)	802 (1.6)	1,421 (1.8)	1,593 (1.6)	830 (0.7)	395 (0.2)	340 (0.2)	266 (0.1)	255 (0.1)

Data are presented as mean (SD) or median (quartile). SD, standard deviation; IQR, Interquartile range.

[a] Only included survivors

Figure legends

Figure S1. Flow chart of the study population

Figure S2. Temporal changes of gross medical costs and number of sepsis patients between 2010 and 2017

The bar plot depicts the relationship between the year of hospital admission on the x-axis and gross medical costs on the y-axis. The line plot depicts another relationship between the admission year and the number of sepsis patients in this cohort on the y-axis.

Figure S3. Annual changes of the medical costs and length of hospital stay according to the sex

(A) Annual changes in unadjusted medical costs per hospitalization between 2010 and 2017 according to the sex. Male: – \$1067/year [95% CI – \$1097 to – \$1037], $p <$ 0.0001. Female: – \$1066/year [95% CI – \$1102 to – \$1031], $p < 0.0001$. The error bars indicate 95% confidence interval. (B) Annual changes in length of hospital stay between 2010 and 2017 according to the sex. Male: – 1.7 [95% CI – 1.8 to – 1.5], $p <$ 0.0001. Female: –1.7 [95% CI –1.8 to –1.6], $p < 0.0001$. The error bars indicate 95% confidence interval. The coefficient was calculated using a linear regression analysis.

Figure S4. Annual changes of the medical costs and length of hospital stay according to the age subgroups

(A) Annual changes in unadjusted medical costs per hospitalization between 2010 and 2017 according to the age subgroups. Adults ($20 \leq$ age ≤ 64): – \$1134/year [95% CI – \$1202 to – \$1066], $p < 0.0001$. Early elderly ($65 \leq$ age ≤ 74): – \$1063/year [95% CI – \$1120 to – \$1007], $p < 0.0001$. Late elderly ($75 \leq$ age): – \$886/year [95% CI – \$908 to – \$864], $p < 0.0001$. (B) Annual changes in length of hospital stay between 2010 and 2017 according to the age subgroups. Adults ($20 \leq$ age ≤ 64): –1.8 [95% CI –2.1 to –1.5], $p < 0.0001$. Early elderly ($65 \leq$ age ≤ 74): –1.8 [95% CI –1.9 to –1.7], $p <$

0.0001. Late elderly (75 ≤ age): –1.5 [95% CI –1.6 to –1.5], $p < 0.0001$. The error bars indicate 95% confidence interval. The coefficient was calculated using a linear regression analysis.

Figure S5. Annual changes of the medical costs and length of hospital stay according to the site of infection

(A) Annual changes in unadjusted medical costs per hospitalization between 2010 and 2017 according to the site of infection. Heart: – $82/year [95% CI – $495 to + $331], $p = 0.69$. Blood: – $2,331/year [95% CI – $3564 to – $1099], $p < 0.0001$. Meninges/brain/spinal cord: – $445/year [95% CI – $684 to – $205], $p < 0.0001$. Unknown: – $1,156/year [95% CI – $1,250 to – $1,062], $p < 0.0001$. Multiple: – $1336/year [95% CI – $1384 to – $1287], $p < 0.0001$. Bone and soft tissue: – $957/year [95% CI – $1072 to – $842], $p < 0.0001$. Abdominal: – $710/year [95% CI – $768 to – $653], $p < 0.0001$. Respiratory: – $865/year [95% CI – $895 to – $835], $p < 0.0001$. Urogenital: – $561/year [95% CI – $621 to – $502], $p < 0.0001$.

(B) Annual changes in length of hospital stay between 2010 and 2017 according to the site of infection. The error bars indicate 95% confidence interval. Meninges/brain/spinal cord: –0.7 [95% CI –1.7 to –0.2], $p < 0.0001$. Blood: –2.8 [95% CI –4.2 to –1.3], $p < 0.0001$. Heart: –0.9 [95% CI –1.5 to –0.3], $p = 0.001$. Unknown: –1.7 [95% CI –2.0 to –1.5], $p < 0.0001$. Bone and soft tissue: –1.8 [95% CI –2.5 $ to –1.2], $p < 0.0001$. Multiple: –2.0 [95% CI –2.1 to –1.8], $p < 0.0001$. Abdominal: –1.2 [95% CI –1.5 to –1.0], $p < 0.0001$. Urogenital: –1.3 [95% CI –1.7 to –0.9], $p < 0.0001$. Respiratory: –1.5 [95% CI –1.6 to –1.4], $p < 0.0001$. The error bars indicate 95% confidence interval. The coefficient was calculated using a linear regression analysis.
